# Various synthesis and biological evaluation of some tri -tetra-substituted imidazoles derivatives: A review

**DOI:** 10.1016/j.heliyon.2024.e31253

**Published:** 2024-05-16

**Authors:** Abdeljalil Hamdi, Walid Daoudi, Mohamed Aaddouz, Mohamed Azzouzi, Hassan Amhamdi, Abdellah Elyoussfi, Abdelmalik El Aatiaoui, Dakeshwar Kumar Verma, Mohamed Abboud, M'hamed Ahari

**Affiliations:** a Applied Chemistry Research Unit, FSTH, Abdelmalek Essaâdi University, AL Hoceima, Tetouan, Morocco; b Laboratory of Molecular Chemistry, Materials and Environment (LCM2E), Departement of Chemistry, Multidisciplinary Faculty of Nador, University Mohamed I, 60700 Nador, Morocco; c Laboratoire de chimie analytique appliquée, matériaux et environnement (LC2AME), Faculté des Sciences, B.P. 717, 60000 Oujda, Morocco; d Department of Chemistry, Government Digvijay Autonomous Postgraduate College, Rajnandgaon, Chhattisgarh-491441, India; e Catalysis Research Group (CRG), Department of Chemistry, College of Science, King Khalid University, Abha 61413, Saudi Arabia

**Keywords:** Tri-tetra-substituted imidazole, Radziszewski reaction, Preclinical approaches, Pharmacological applications

## Abstract

The imidazole nucleus represents a significant group of heterocyclic molecules with diverse significance in the modern world due to its exploration potential and various pharmacological applications. The relevance of imidazole and its derivatives has gained popularity in recent years, especially in the production of commercial drugs and the treatment of various conditions. The imidazole nucleus is present in many natural compounds and widely distributed in essential amino acids, such as l-histidine, whose derivatives exhibit powerful pharmacological properties. In this review, we delve into the historical timeline and development of synthetic pathways for tri- and tetra-substituted imidazoles used in the renowned *Radziszewski* reaction. Furthermore, we explore various bacteriological applications documented in the literature, as well as current advances in preclinical approaches to imidazole-based drug discovery. Tri- or tetra-substituted imidazole derivatives show strong potential for new synthesis methods, such as reflux or microwave, as well as various biological activities.

## 1 Introduction

Imidazole is a planar five-membered heterocycle with the typical chemical formula (CH)_2_N(NH)CH, containing two double bonds, two nitrogen atoms, three carbon atoms, and four hydrogen atoms. It is a colorless solid that dissolves in water and other polar solvents. In chemistry, it is classified as an aromatic heterocycle belonging to the diazole and alkaloid families. Imidazole is amphoteric, meaning it can act as both a base and an acid [[1], [2], [3]]. The molecule is considered aromatic due to the existence of a sextet of ***π*** electrons, comprised of a protonated nitrogen atom sharing a pair of electrons with one electron from each of the other four atoms in the ring. Heterocyclic structures play a crucial role in organic chemistry research and development, with millions of them having been discovered, each possessing unique characteristics and biological importance. Imidazole has been deliberately chosen from a range of chemicals due to its interesting biomedical activity and ability to form complexes with certain dyes [[4], [5], [6], [7], [8]]. For instance, Al-Adilee et al. [6] synthesized metal complexes for Pd(II) and Pt (IV) ions with an imidazole core for antioxidant and therapeutic applications. Their study demonstrated that the anticancer activity of imidazole complexes against breast cancer receptors was associated with their interaction with BCL-2 and a BAX BH3 peptide. Similarly, another study focused on preparing a series of metal complexes of imidazoles with the Ag(I) ion for biological applications [9]. The authors found that antimicrobial tests revealed significantly higher activity of Ag (I) complexes against fungi compared to bacterial species, highlighting the significant importance of imidazole metal complexes in the pharmacological field. It is conceivable that isomeric versions of imidazole, with a free imino hydrogen and a substituent in positions 4 and 5, or with two different substituents in these locations, may exist. The position of the imino hydrogen, which can be attached to either one of the two positively charged nitrogen atoms or both depending on the shape of the tautomer, varies [10]. Imidazole has emerged as a key component in the development of novel pharmaceutical medications. Heterocyclic imidazole derivatives exhibit a multitude of biological activities, including antibacterial properties [11], anti-inflammatory effects [12], antimycobacterial, anti-allergic, antitumor [13], antidiabetic [14], antiviral [15], antioxidant [16], antimalarial [17], anthelmintic, antifungal, and ulcerogenic activities [18].

**Scheme 1:**
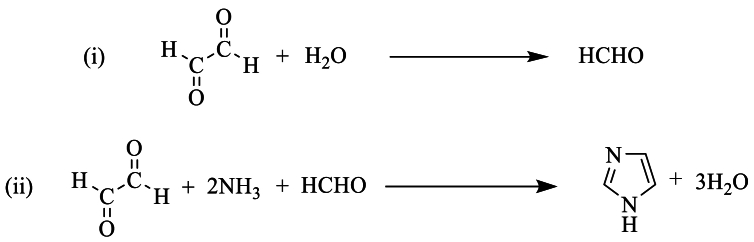

This review encompasses various synthesis pathways for tri- and tetra-substituted imidazoles, starting from benzyl, 9,10-phenanthrenequinone, isatin, acenaphthenequinone, and tracing their synthesis from discovery to the final product. The review then highlights different synthetic methodologies documented in the literature. One such method involves reacting ammonia with glyoxal to produce imidazole. However, the mechanism of this reaction has remained unknown, although one theory suggests that one molecule of glyoxal degrades into formic acid and formaldehyde. The latter then reacts as illustrated in Scheme 1 [19].

## 2 Major synthetic procedures

**Scheme 2:**
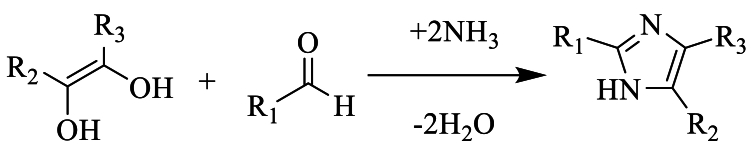

Heinrich Debus et al. have synthesized imidazole for the first time in 1858 [20], by ammonia combining glyoxal and formaldehyde, to form imidazole as indicated in the diagram below (Scheme 2).

**Scheme 3:**
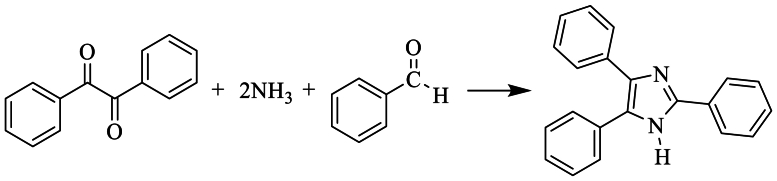

Radiszewski et al. have identified triphenyl imidazole from condensation of benzyl such as di-carbonyl or glyoxal compound with benzaldehyde, α-keto aldehyde in the presence two ammonia molecules, almost after a one hundred years (Scheme 3) [21].

**Scheme 4:**
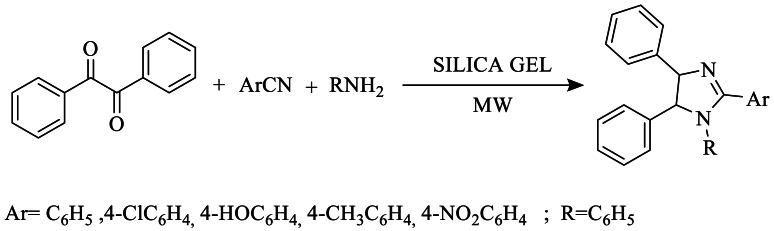

However, Bourissou et al. [22] synthesized the 1-substituted, 2-aryl, 4,5-phenylimidazole using benzyl, benzonitrile, and primary amines on the surface of silica gel under solvent-free conditions with microwave irradiation, yielding moderate efficiencies (Scheme 4).

**Scheme 5:**
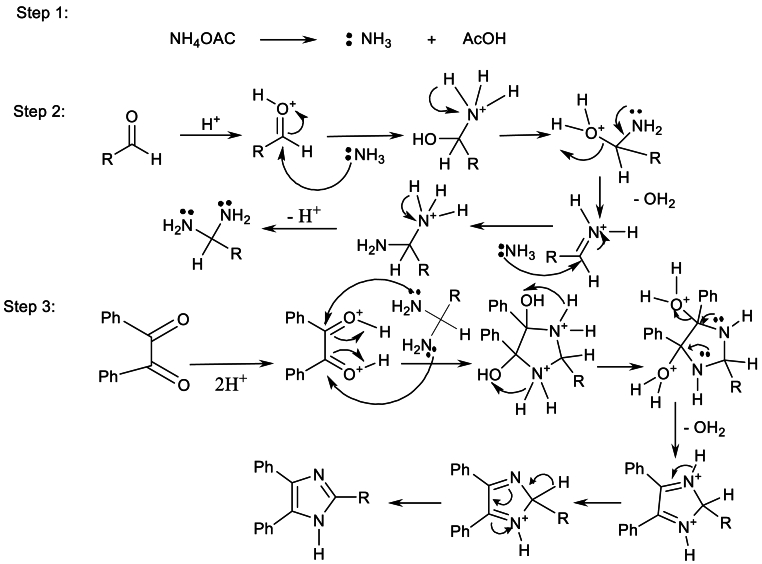

The mechanism of the *Debus-Radziszewski* reaction for the synthesis of 2,4,5-trisubstituted imidazole is provided in Scheme 5 [23].

**Scheme 6:**
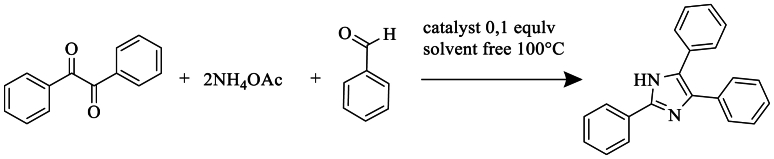

Safari et al. [24] obtained trisubstituted imidazoles in 80 % yields using the condensation of benzyl, various aldehydes and NH_4_OAc under microwave irradiation, under solvent-free conditions. The effective catalyst employed was (NH_4_)_6_Mo7O_24_.4H_2_O (Scheme 6).

**Scheme 7:**
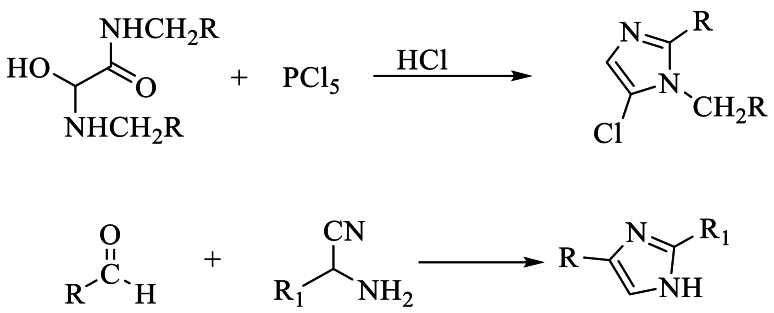

Wallach et al. [25] have reported the reaction of N, N-dimethyl oxamide with phosphorus pentachloride PCl_5_ as a convenient method of obtaining 5-chloro-substituted chlorinated imidazole in extremely moderate yields. Imidazole can be obtained by reacting aldehydes and aminonitrile derivatives (Scheme 7).

**Scheme 8:**
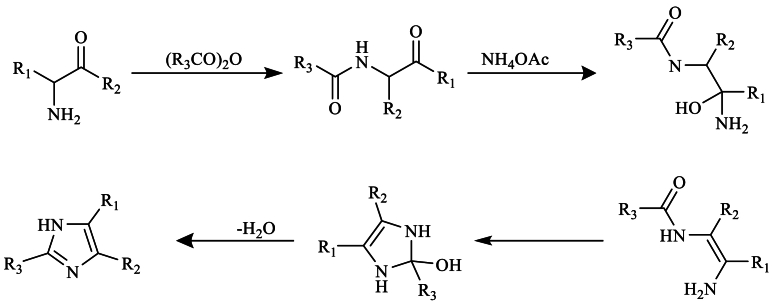

Moreover, cyclization of α-acylaminoketones in the presence of ammonium anhydride and acetate leads to the formation of imidazole in excellent yields (Scheme 8) [26].

## 3 The development of derivatives tri-or tetra imidazole, via microwave processes

**Scheme 9:**
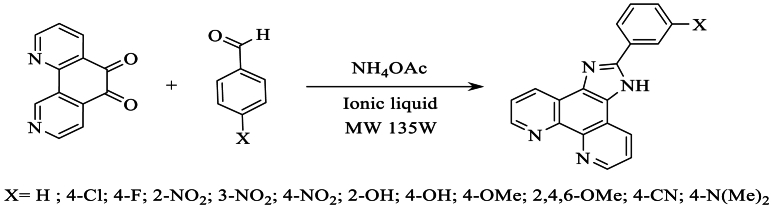

Qasim et al. [27] conducted the synthesis of 2-phenylimidazo [4,5-f], [1,10]phenanthrolines in a single step from three components: 1,10-phenanthroline-5,6-dione, various aldehydes, and NH_4_OAc, using SnCl_2_.2H_2_O as a cost-effective and non-hazardous catalyst. This reaction was carried out at room temperature, resulting in an excellent yield of 85 %, as illustrated in Scheme 9.

**Scheme 10:**
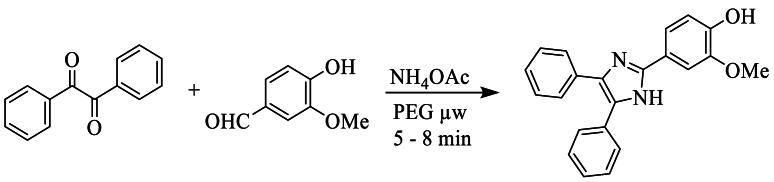

In 2010, Nalage et al. [28] carried out the synthesis of triaryl-imidazole in the presence of polyethylene glycol using benzyl, 3-methoxy-4-hydroxybenzaldehyde, and ammonium acetate under microwave irradiation for 5 min, achieving yields of up to 71 % (Scheme 10).

**Scheme 11:**
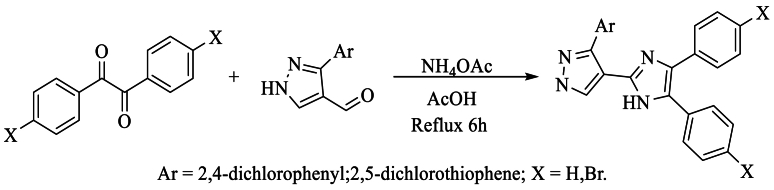

However, Wahyuningrum et al. [29] conducted the synthesis of 4,5-disubstituted imidazoles in the presence of MAOS by reacting a diketone with various aldehydes or ketones, using NH_4_OAc in glacial AcOH assisted by microwave irradiation for 5–7 min, achieving yields exceeding 84 % (Scheme 11).

**Scheme 12:**
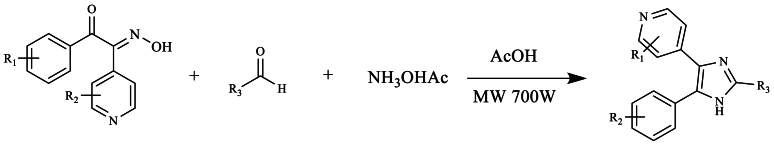

Furthermore, Sparks et al. [30] synthesized the tri-substituted imidazole using keto-oxime and aldehyde with NH_4_OAc in AcOH under microwave irradiation, with yields not exceeding 70 % during the cyclization of N-hydroxy imidazole at around 200 °C for 20 min (Scheme 12).

## 4 Other techniques for synthesizing tri or tetra - imidazole include

**Scheme 13:**
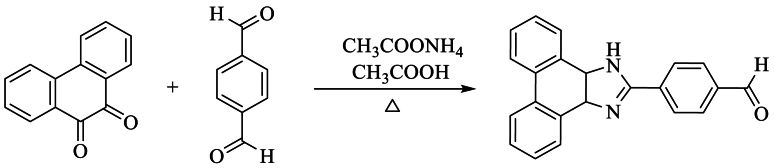

This section of the review aims to explore alternative methods for synthesizing 2,4,5-substituted imidazoles, as well as 1,2,4,5-tetrasubstituted imidazoles. Consequently, several research groups have dedicated themselves to the synthesis of imidazole and the investigation of its numerous pharmacological properties. Steck and Day et al. synthesized 4-(1H-phenanthro [9,10-d]-imidazole-2-yl)-benzaldehyde from 9,10-phenanthroquinone with terephthaldehyde and NH_4_OAc in glacial AcOH, achieving excellent yields exceeding 70 % (Scheme 13) [31].

**Scheme 14:**
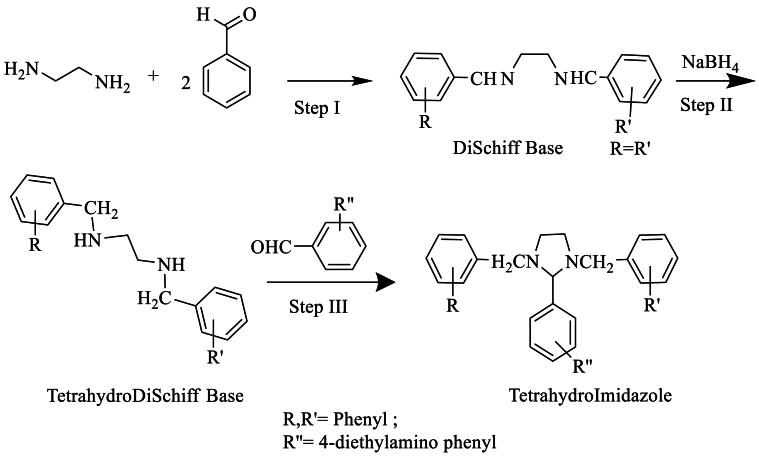

In 2001, Sharma et al. [32] synthesized their respective Schiff bases from aromatic aldehydes using ethylenediamine. These new compounds were then reduced to obtain tetrahydrodi Schiff bases. Finally, these derivatives were prepared with various aromatic aldehydes to yield tetrahydroimidazoles with yields exceeding 80 % (Scheme 14).

**Scheme 15:**
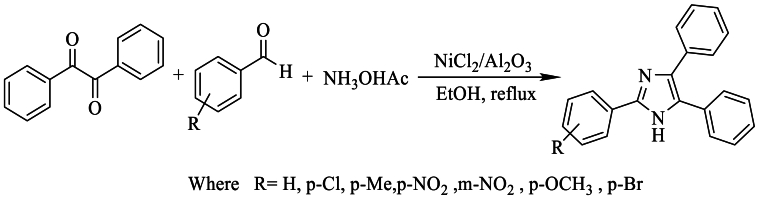

In 2007, Heravi et al. [33] synthesized triphenyl-imidazole with yields exceeding 92 % by condensing benzyl acetate and ammonium with aldehydes, in the presence of NiCl_2_.6H_2_O as a catalyst in refluxing ethanol (Scheme 15).

**Scheme 16:**
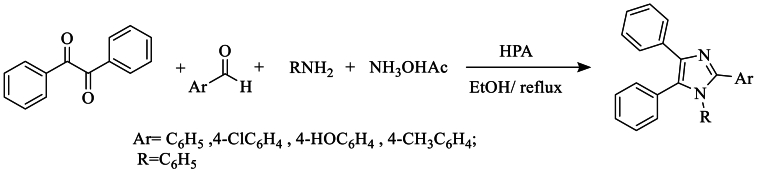

The same year, they developed and improved an efficient procedure for the synthesis of a tetrasubstituted imidazole, using Keggin-type heteropolytungstic acid (HPA) as a catalyst in refluxing ethanol (Scheme 16).

**Scheme 17:**
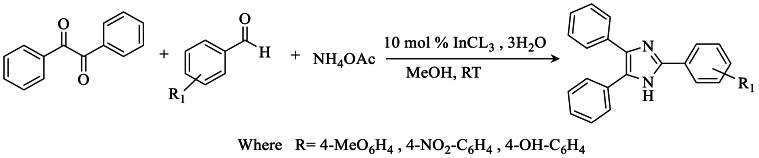

In 2008, Sharma et al. [34] accomplished the one-step synthesis of tri-substituted or tetra-substituted imidazoles with yields reaching 82 % at room temperature. This synthesis was carried out using benzyl, NH_4_OAc in the presence of InCl_3_.3H_2_O as a catalyst, and benzaldehyde (Scheme 17).

**Scheme 18:**
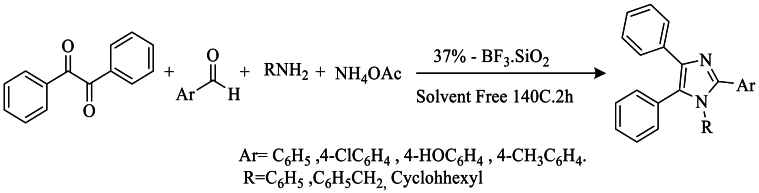

That year, Sadeghi et al. [35] synthesized 1,2,4,5-tetra-substituted imidazole using silica-supported boron trifluoride (BF_3_, SiO_2_) as a catalyst, a reusable and cost-effective material, in reaction with benzyl, aromatic aldehyde, amine, and NH_4_OAc. This one-step reaction is very simple, rapid, efficient, and yields a very high yield, reaching 96 % (Scheme 18).

**Scheme 19:**
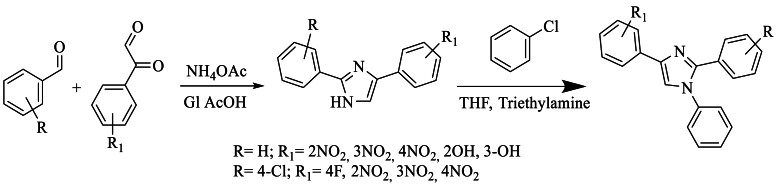

In 2009, Husain et al. [36] synthesized disubstituted imidazole using aromatic aldehydes, phenylglyoxal, ammonium acetate, and acetic acid. Subsequently, by mixing the 1,2,4-disubstituted imidazoles with chlorobenzene in the presence of triethylamine and tetrahydrofuran (THF), the new product was obtained with a yield of 74 % (Scheme 19).

**Scheme 20:**
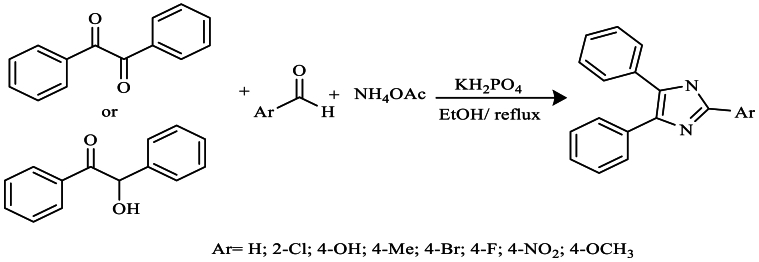

In 2010, Joshi et al. [37] synthesized 2-aryl, 4,5-diphenylimidazole by refluxing it in ethanol for 40–90 min under mild reaction conditions. They employed benzyl/benzoin with various aldehydes and ammonium acetate. This method, conducted in the presence of potassium dihydrogen phosphate (KH_2_PO_4_), is simple, effective, and resulted in excellent yields reaching 93 % (Scheme 20).

**Scheme 21:**
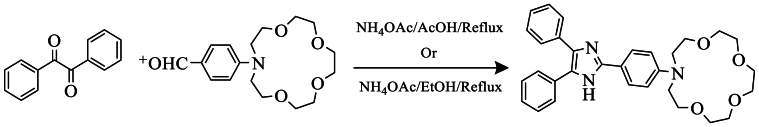

In the same year, Oliveira et al. [38] prepared a new family of Diphenyl-imidazole-2-yl-phenyl-tetraoxa7-azacyclopentadecane from the formyl azacrown ether with NH_4_OAc in glacial AcOH or ethanol for 12 h at reflux. This straightforward reaction resulted in a good to excellent yield, exceeding 64 % (Scheme 21).

**Scheme 22:**
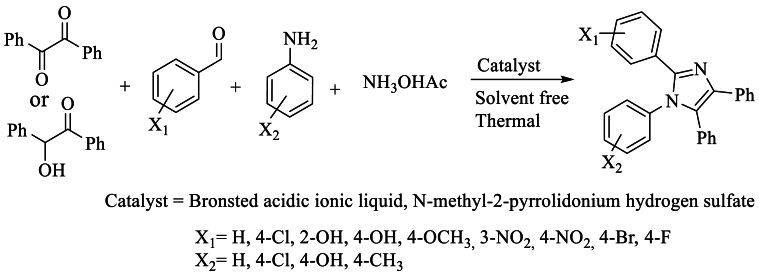

In 2011, Shaterian and Runge et al. [39] achieved the synthesis of 2,4,5-substituted imidazoles, as well as 1,2,4,5-tetrasubstituted imidazoles, using a solvent-free ionic liquid and a catalyst containing a Brønsted acid, namely triphenyl (propyl-3-sulfonyl) phosphonium toluene sulfonate, at a temperature of 100 °C. This method yielded excellent yields surpassing 96 % (Scheme 22).

**Scheme 23:**
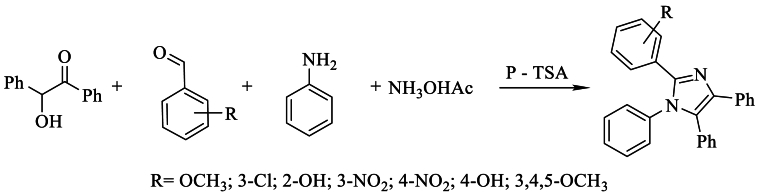

In the same year, *Pasha* et al. [40] developed the synthesis of 1,4,5-phenyl,2-substituted imidazoles by condensing benzoin, aniline, NH_4_OAc, and araldehydes with a simple catalyst, *p*-toluenesulfonic acid, in ethanol as a solvent under reflux. This method is straightforward, rapid, and resulted in yields of up to 92 % (Scheme 23).

**Scheme 24:**
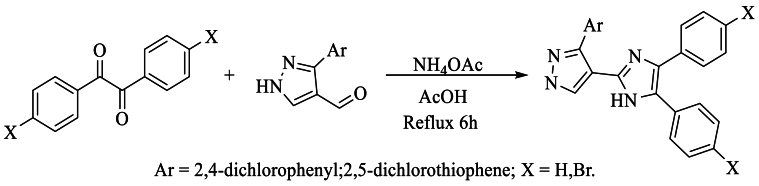

Furthermore, Vijesh et al. [41] synthesized 2,4,5-trisubstituted compounds by reacting 3-aryl-1H-pyrazole-4-carbaldehyde and 2-diketones with NH_4_OAc in the presence of glacial acetic acid under reflux, achieving reasonably high yields up to 86 % (Scheme 24).

**Scheme 25:**
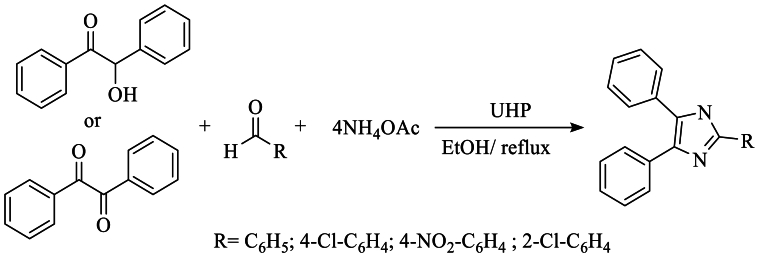

In 2013, Maleki et al. [42] synthesized 2-substituted 4,5-diphenylimidazole using benzyl or benzoin, NH_4_OAc, and aldehydes, with hydrogen peroxide (UHP) as the catalyst, in ethanol under reflux. This reaction resulted in a yield of 88 %, as illustrated in Scheme 25.

**Scheme 26:**
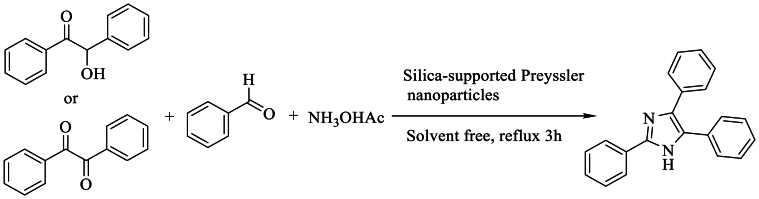

In 2014, Gharib et al. [43] accomplished the multicomponent synthesis of 2,4,5- and 1,2,4,5-tetrasubstituted -1H-imidazole derivatives through the condensation of benzyl/benzoin, NH_4_OAc, and aldehydes in the presence of silica-supported heteropolytungstic acid as a catalyst, with reflux, resulting in an excellent yield exceeding 94 % (Scheme 26).

**Scheme 27:**
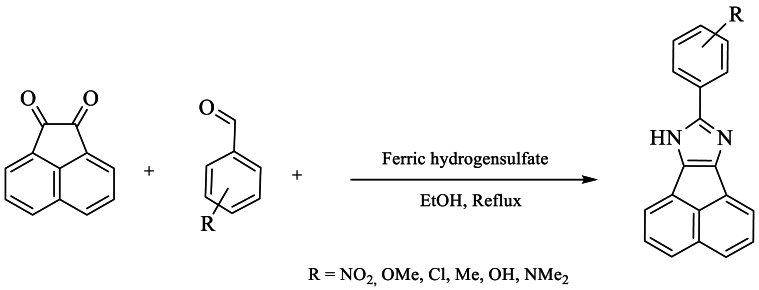

In the same year, *Sandroos* et al.*l* [44] developed an innovative and efficient method for the synthesis of 8-aryl-7H-acenaphtho [1,2-d]imidazole, resulting in excellent yields of 93 % under reflux. This approach involved the use of acenaphthylene-1,2-dione, substituted aldehydes, NH_4_OAc, and ericinium hydrogen sulfate in ethanol (Scheme 27).

**Scheme 28:**
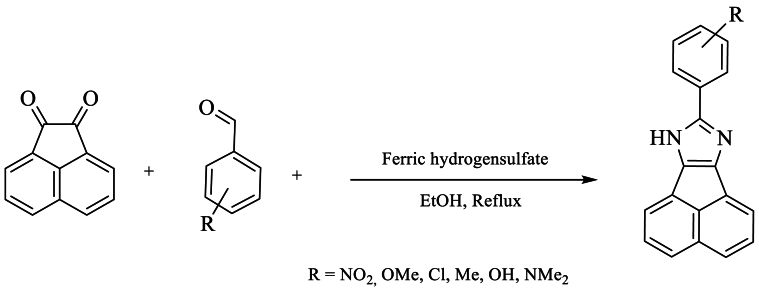

In 2015, Subeesh et al. [45] synthesized 2-(pyren-1-yl)-1H-phenanthro [9,10-d]imidazole with a yield of 60 %. This synthesis was carried out using phenanthrene-9,10-dione and pyrene-1-carbaldehyde with NH_4_OAc in the presence of glacial AcOH under a nitrogen atmosphere, during a 12-h reflux (Scheme 28).

**Scheme 29:**
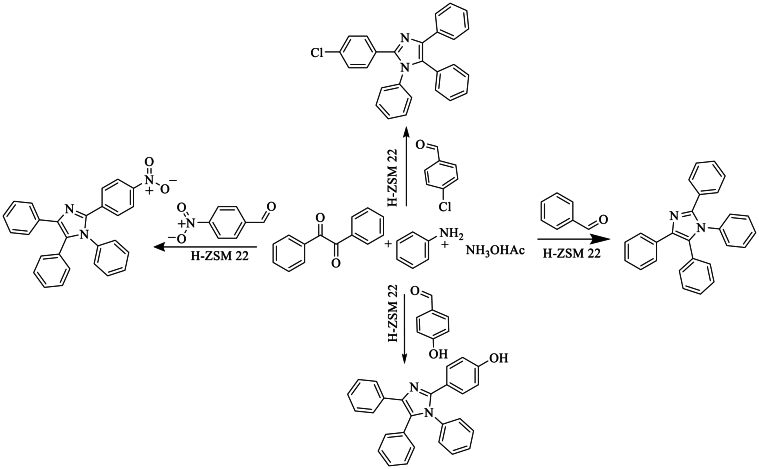

In 2016, Bha et al. [46] synthesized tetra-substituted imidazoles using various aldehyde derivatives in this reaction, with benzyl, amines, and NH_4_OAc in the presence of H-ZSM-22 as a catalyst, achieving a good yield of 86 % under solvent conditions (Scheme 29).

**Scheme 30:**
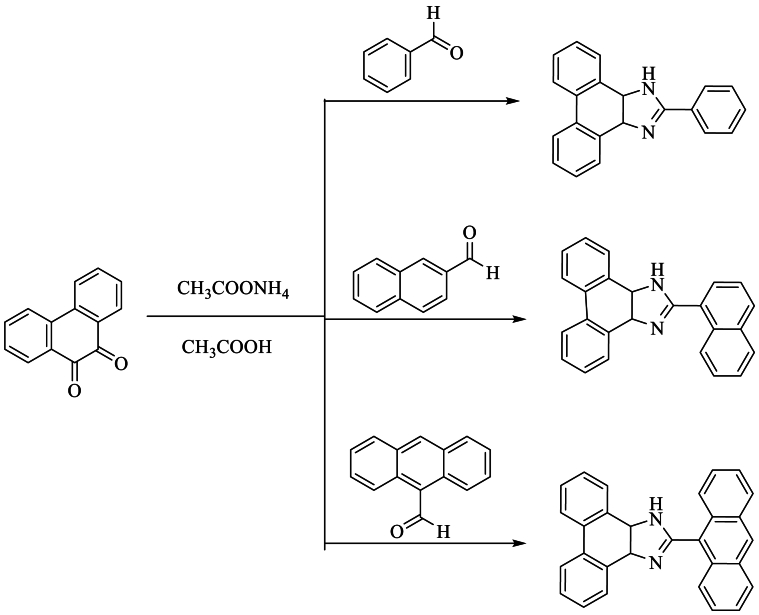

In the same year, Wang et al. [47] synthesized and characterized three ligands: 2-phenyl-1H-imidazo [4,5-f], [1,10]phenanthroline from benzaldehyde (1), 2-(2-naphthyl)1H-imidazo [4,5-f]phenanthroline from 2-naphthaldehyde (2), and 2-(2-anthryl)-1H-imidazo [4,5-f]phenanthroline from 9-anthrylaldehyde (3). These syntheses were carried out in the presence of ammonium acetate and AcOH for 4 h under reflux, with yields ranging from 45 to 68 % (Scheme 30).

**Scheme 31:**
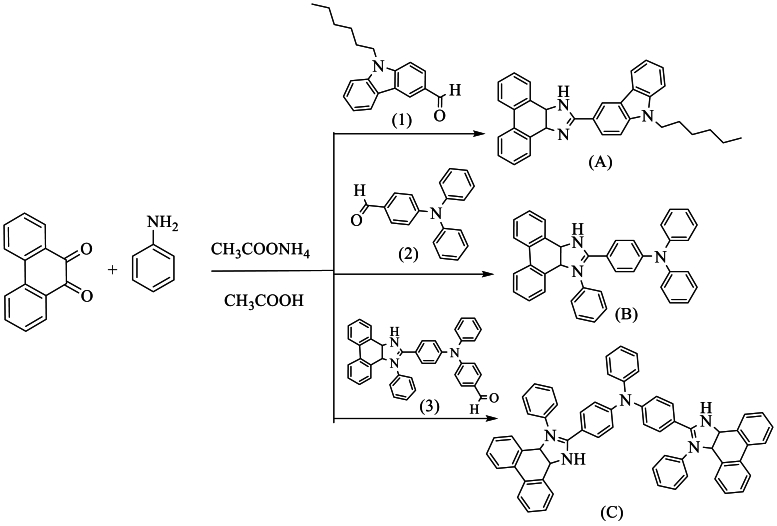

Additionally, Tavgeniene et al. [48] described three new derivatives, namely 2-(9-hexylcarbazolyl-3-yl)-1-phenylphenanthro [9,10-d]imidazole (A), 2-[4-(N,N-diphenylamino)phenyl]-1-phenylphenanthro [9,10-d]imidazole (B), and bis [4-(1-phenylphenanthro [9,10-d]imidazole-2-yl)phenyl]-N-phenylamine (C). These compounds were obtained using an excess of phenanthro [9,10-d]imidazole and 3-formyl-9-hexylcarbazole (1), 4-(diphenylamino)benzaldehyde (2), and diformyltriphenylamine (3), with NH_4_OAc and aniline in AcOH, resulting in yields ranging from 64 to 44 % (Scheme 31).

**Scheme 32:**
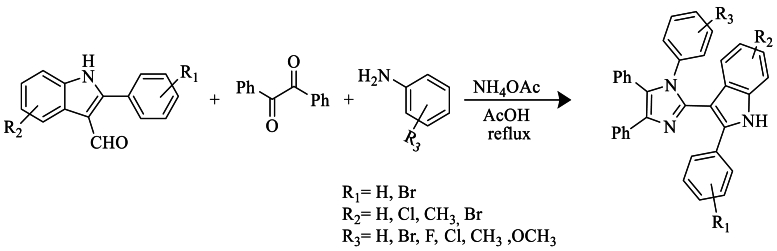

In 2017, Naureen et al. [49] synthesized tetra-arylimidazole based on indole by refluxing, using 2-arylindole-3-carbaldehydes, benzyl, anilines, and NH_4_OAc in the presence of AcOH (Scheme 32).

**Scheme 33:**
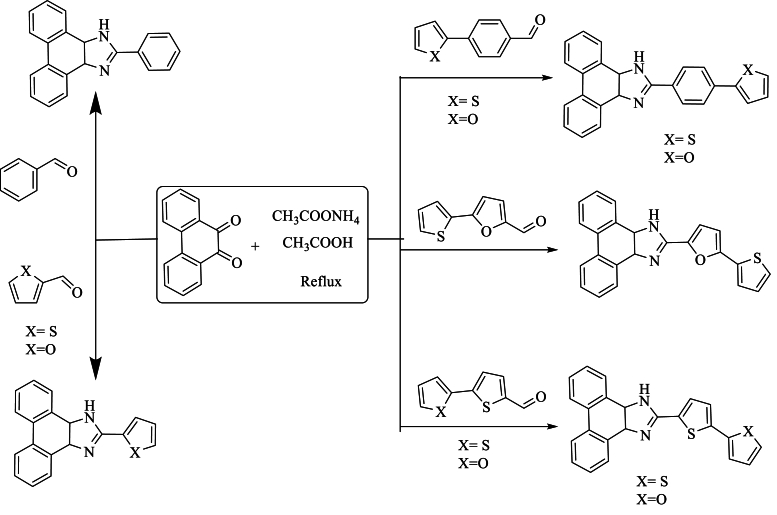

In the same year, Ferreira et al. [50] synthesized phenanthroimidazole ligands using the Radziszewski method, employing various synthetic methodologies involving the precursors 9,10-phenanthrenedione and formaldehyde with NH_4_OAc in glacial AcOH. This synthesis was carried out with moderate to excellent yields, ranging from 54 to 89 %, by refluxing for 8 h (Scheme 33).

**Scheme 34:**
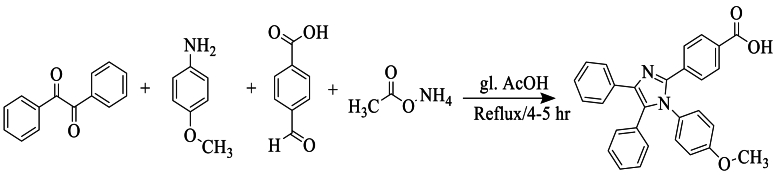

In 2018, Ravindra et al. [51] synthesized a new compound, 4-(1-(4-methoxyphenyl)-4,5-diphenyl-1H-imidazole-2-yl)phenyl carboxylic acid, from 4-methoxyaniline, benzyl, NH_4_OAc, and 4-formyl-benzoic acid, using glacial acetic acid as a solvent and a catalytic amount of concentrated sulfuric acid, under reflux, yielding 70 % (Scheme 34).

**Scheme 35:**
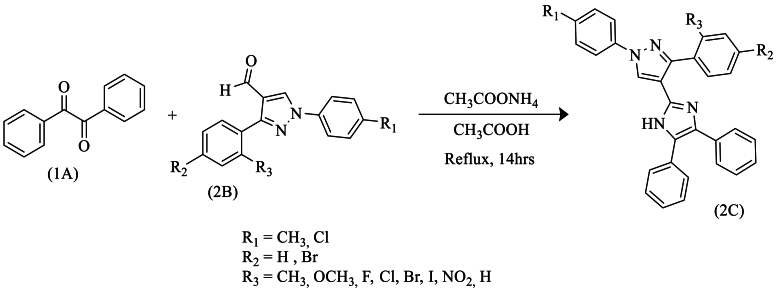

In the same year, Harshad et al. [52] prepared new tri-substituted imidazole derivatives (2C) using the well-known Radziszewski reaction. This synthesis was based on different derivatives of 1H-pyrazole-4-carbaldehyde (2B), with benzyl (1A) and NH_4_OAc in glacial acetic acid, achieving good to excellent yields under reflux conditions (Scheme 35).

**Scheme 36:**
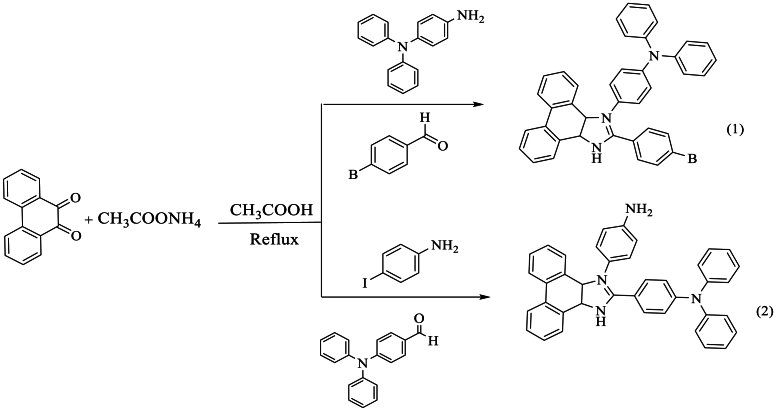

Similarly, Anupama et al. [53] developed a compound (1) synthesized from 9,10-phenanthrenequinone with bromobenzaldehyde and N1,N1-diphenylbenzene-1,4-diamine. For compound (2), it was prepared from 9,10-phenanthrenequinone with (diphenylamino)benzaldehyde and 4-iodoaniline. In both cases, the mixture with NH_4_OAc in the presence of glacial AcOH for 4 h at reflux resulted in respective yields of 68 % and 76 % (Scheme 36).

**Scheme 37:**
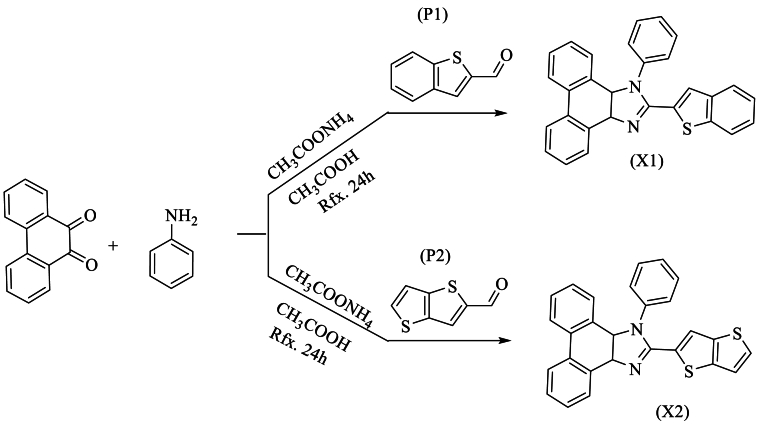

Additionally, *Kula* et al. [54] synthesized a new compound, phenanthro [9,10-d]imidazole, with an excellent yield ranging from 76 % to 72 %. This cost-effective synthesis was achieved through an efficient method, the Debus Radziszewski reaction, by combining benzo [*b*]thiophene-2-carbaldehyde (P1) or thieno [3,2-b]thiophene-2-carbaldehyde (P2) with phenanthrenequinone, aniline, and NH_4_OAc in the presence of acetic acid to obtain the new compound 2-(benzo[*b*]thiophene-2-yl)-1-phenyl-1H-phenanthro [9,10-d]imidazole (X1) or 2-(thieno [3,2-b]thiophene-2-yl)-1-phenyl-1H-phenanthro [9,10-d]imidazole (X2) (Scheme 37).

**Scheme 38:**
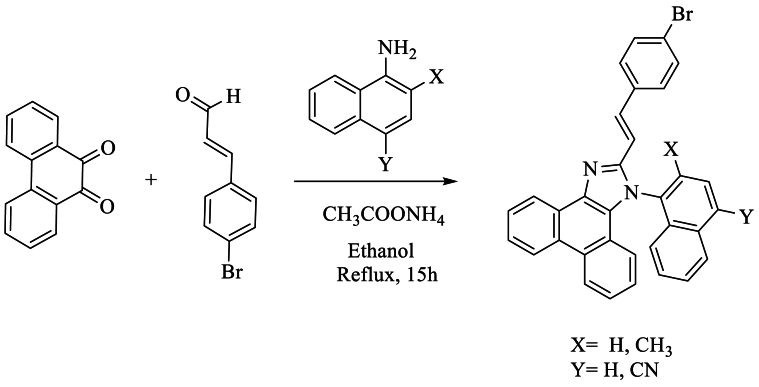

However, Jayaraman et al. [55] synthesized 2-(4-bromostyryl)-1-(naphthalen-1-yl)-1H-phenanthro [9,10-d]imidazole using phenanthrene-9,10-dione, 4-bromo-1-cinnamaldehyde, 1-naphthylamine, and NH_4_OAc in AcOH or ethanol, refluxing for approximately 15 h, and achieving yields ranging from 58 % to 68 % (Scheme 38).

**Scheme 39:**
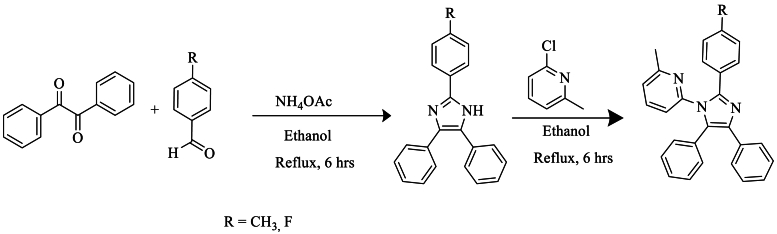

In 2019, Amala et al. [56] utilized the Radziszewski reaction as the foundation for the synthesis of derivatives of 2,4,5-triphenyl-1H-imidazole-1-(2-chloro)-6-methylpyridine. They carried out this synthesis in the presence of ethanol as the solvent, with anhydrous potassium carbonate and 2-chloro-6-methylpyridine, stirred at cold temperatures for 6 h, achieving a good yield ranging between 87 and 90 % (Scheme 39).

**Scheme 40:**
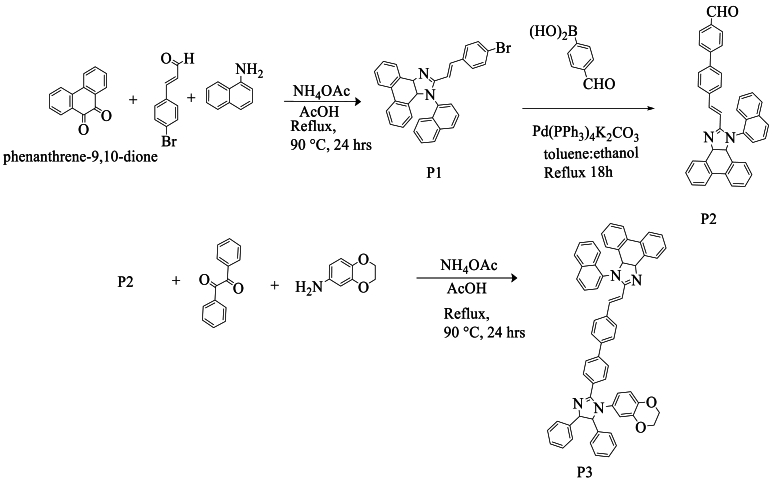

In the same year, Jayabharathi et al. [57] described a new protocol for the synthesis of compound (P1) with a yield of 75 %. This compound was synthesized from 9,10-phenanthrenequinone, naphthalene-1-amine, and (E)-3-(4-bromophenyl)acrylaldehyde in the presence of ammonium acetate in acetic acid under a nitrogen atmosphere. Then, by mixing the obtained product with 4-formylphenylboronic acid, aqueous K_2_CO_3_ in a toluene:ethanol mixture, using Pd(PPh_3_)_4_ as a catalyst, the product was obtained with a yield of 73 %. For the resulting compound (P2), a mixture with 2,3-dihydrobenzo [b] [1,4]dioxine-6-amine and benzyl in acetic acid in the presence of ammonium acetate was refluxed, resulting in the final product (P3) with a good yield ranging between 62 and 78 % (Scheme 40).

**Scheme 41:**
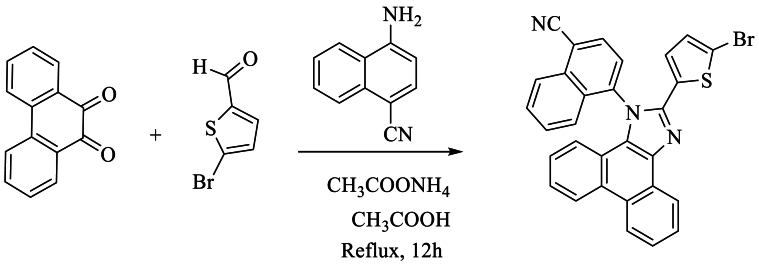

However, Jayaraman et al. [58] have synthesized new compound of the imidazole [4-(2-(5-bromothiophen-2-yl)-1H-phenanthro [9,10-d]imidazole-1-yl)naphthalene-1-carbonitrile] with good yield 65–70 % by using 5-bromothiophene-2-carbaldehyde, 4-aminonaphtalene-1-carbonitrile, and NH_4_OAc in AcOH at reflux about 12h (Scheme 41).

**Scheme 42:**
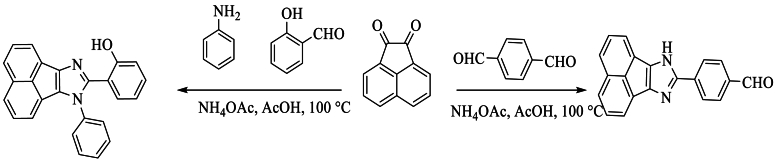

On the other hand, Singh et al. [59] prepared the products 2-(7-phenyl-7H-acenaphtho [1,2-d]imidazole-8-yl)phenol (A) and 4-(7H-acenaphtho [1,2-d]imidazole-8-yl)benzaldehyde (B) in 52–64 % yield from the condensation of acenaphthoquinone and aniline, salicylaldehyde for compound (A), but terephthaldehyde for compound (B), all reagents being used with NH4OAc in glacial AcOH (Scheme 42).

**Scheme 43:**
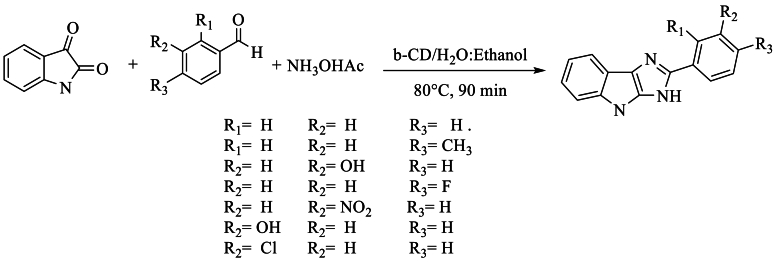

In 2020, Nipate et al. [60] synthesized 1,8-dihydroimidazo [2,3-b]indoles with yields of 79 % using β-cyclodextrin (β-CD) as an efficient, biodegradable, and recyclable catalyst. The reaction was carried out with isatin and three aldehyde compounds in the presence of NH_4_OAc in a H_2_O–EtOH mixture under reflux. This method exhibited high yield, short reaction time, cost-effective starting material, non-toxicity, environmental friendliness, and easy availability (Scheme 43).

**Scheme 44:**
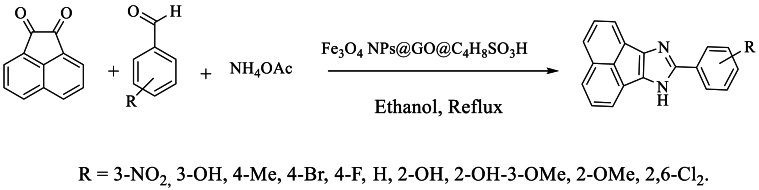

In the same year, Hasanzadeh et al. [61] prepared 8-aryl-7H-acenaphtho [1,2-d]imidazole using Fe_3_O_4_ NPs@GO@C_4_H_8_SO_3_H nanoparticles as a magnetic nanocatalyst, with acenaphthenequinone and ammonium acetate in ethanol under reflux, achieving a high yield ranging between 84 and 95 % (Scheme 44).

**Scheme 45:**
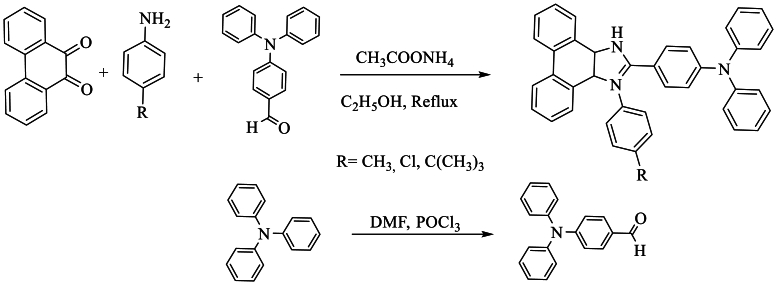

However, Peng et al. [62] prepared a new product (R-PPIM-TPA), where R represents Me, Cl, C(CH_3_)_3_, by combining a solution of 9,10-phenanthrenequinone, 4-(diphenylamino)benzaldehyde, substituted aniline, and NH_4_OAc in ethanol at 100 °C for 8 h, obtaining yields ranging from 51 to 63 % (Scheme 45).

**Scheme 46:**
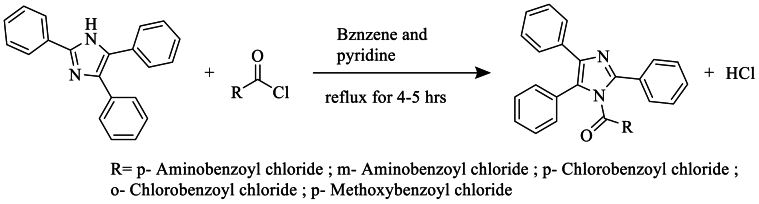

In 2021, Burungale et al. [63] synthesized 1-substituted 2,4,5-triphenylimidazoles using acid chloride and triphenylimidazole in pyridine as a catalyst, with benzene as the solvent, under reflux for 4–5 h. The resulting new compound was isolated with a good yield of 88 % after drying and recrystallization from ethanol (Scheme 46).

**Scheme 47:**
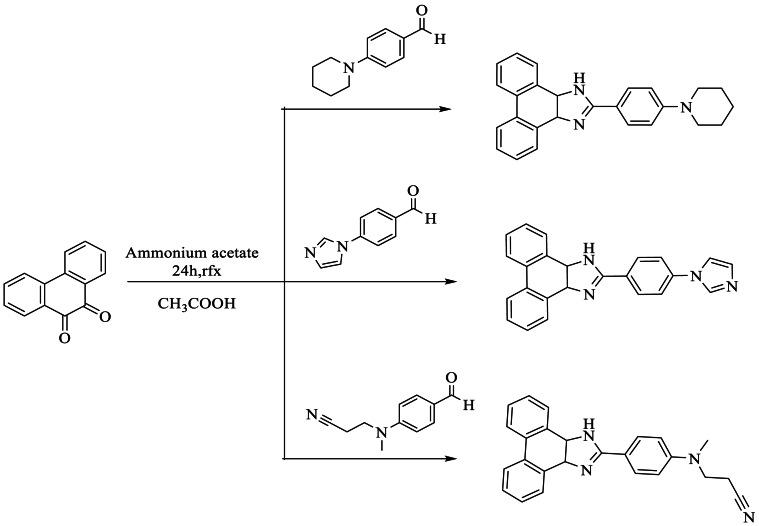

Furthermore, Kula et al. [64] prepared three new compounds using a simple reflux method, achieving a high yield ranging between 61 and 70 %. This was accomplished by condensing 9,10-phenanthrenequinone with an aldehyde (4-(piperidin-1-yl)benzaldehyde, 4-(1H-imidazole-1-yl)benzaldehyde, 4-[(2-cyanoethyl)methylamino]benzaldehyde) and NH_4_OAc in acetic acid (Scheme 47).

## 5 Pharmacological activity

Numerous imidazole synthesis techniques, as well as their diverse structural reactions, in medicinal chemistry, have a wide range of applications. Imidazole and its derivatives, as revealed by literature survey revealed are believed to have anti-anthelmintic activity [65], cardiovascular activity [66], anti-inflammatory and activity [67], anti-neoplastic activity [68], anti-fungal activity [69], enzyme inhibition activity [70], anti-viral activity and anti-ulcer activity [71]. Therefore, Imidazole has various uses in the development of pharmacological and biochemicals [72]. Intimidate, a hypnotic drug, and proton push inhibitor (omeprazole) [73] as well as a benzodiazepine antagonist, is a derivative of the imidazole. The synthesis of imidazole has been a very important goal in recent years for all of the aforementioned applications.

### 5.1 Various pharmacological activities for imidazole derivatives

#### 5.1.1 Antifungal activities

Imidazole derivatives exhibit interesting antifungal activity, making them compounds of great interest in the field of medical research. Table 1 consolidates the antifungal activity of certain imidazole derivatives.

**Table 1:** Antifungal activities of certain imidazole derivatives against three phytopathogenic fungi.

Compounds	bacterial strain	IC_50_ (μg/ml)	Ref.
	*Fusarium oxysporum*	35	[74]
	*Candida albicans*	0.3	
	*Aspergillus niger*	45	
	*Fusarium oxysporum*	20	[74]
	*Candida albicans*	0.25	
	*Aspergillus niger*	40	
	*Fusarium oxysporum*	20	[74]
	*Candida albicans*	0.2	
	*Aspergillus niger*	40	
	*Fusarium oxysporum*	13	[75]
	*Fusarium oxysporum*	12	[76]
	*Candida albicans*	3.125	[77]
	*Candida albicans*	1.56	[77]
	*Candida albicans*	12.5	[78]
	*Aspergillus niger*	25	
	*Candida albicans*	25	[78]
	*Aspergillus niger*	50	
	*Candida albicans*	12.5	[78]
	*Aspergillus niger*	12.5	
	*Aspergillus niger*	200	[79]

**Fig. 1:**
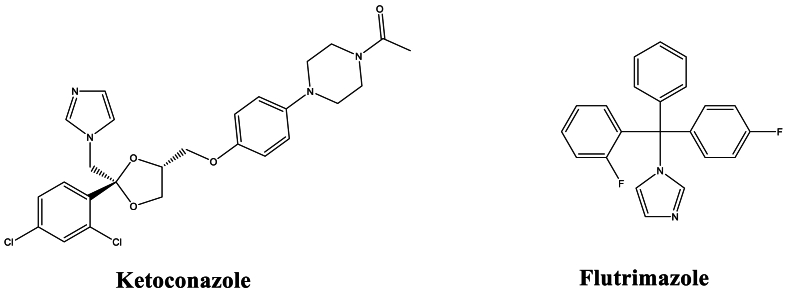
Imidazole antifungal drugs.

The results of Table 1 indicate that imidazole derivatives exhibit interesting antifungal activities against *Fusarium oxysporum, Candida albicans,* and *Aspergillus niger strains*. Specifically, the three imidazole-thiazole derivatives mentioned in Table 1 demonstrated extremely high antifungal activity against *C. albicans, F. oxysporum,* and *A. niger* strains, compared to standard imidazole-based medications (Fig. 1). Nikalje et al. [74] observed that imidazole-thiazole compounds act by inhibiting ergosterol biosynthesis in C. albicans. Similarly, Husain et al. [78] revealed that imidazole derivatives exhibit excellent antifungal activity against C. albicans and A. niger strains with lower gastrointestinal irritation, especially compounds with methoxy (-OCH_3_) and nitro (-NO_2_) groups in the para position.

However, Ghorbani-Vaghei et al. [76] endeavored to investigate novel antifungal agents through in vitro susceptibility tests to establish guidelines for the potential clinical application of these new compounds against the Fusarium oxysporum strain. Both tested compounds exhibited antifungal activity against *Fusarium oxysporum*, especially the one containing an alkyl group in the para position. Consequently, they can be regarded as promising antifungal agents.

#### 5.1.2 Antibacterial activity

The antibacterial properties of imidazole as a compound are well-known. However, its direct use is often restricted due to its toxicity. To enhance both effectiveness and tolerance, modifications are frequently made to imidazole derivatives. Although imidazoles are primarily recognized for their antifungal properties, their antibacterial activity can vary depending on the specific compound and the class of targeted organisms. Table 2 presents some imidazole derivatives and their antibacterial activities against two strains, *Escherichia coli (E. coli)* and *Staphylococcus aureus (S. aureus).*

**Table 2:** The effect of various imidazole derivatives on two antibacterial strains.

Compounds	IC_50_ (μg/ml)		Ref.
	E. coli	S. aureus	
	50	50	[80]
	50	50	[80]
	1000	16	[81]
	32	4	[81]
6	50	50	[82]
	50	50	[82]
	50	50	[82]
	7.8	1.9	[83]
	15.6	31.2	[83]

The results from Table 2 highlight that most derivatives of the imidazole core exhibit promising antibacterial activities, particularly against E. coli and S. aureus strains. Indeed, the presence of the nitro group (-NO_2_) in the compounds listed in Table 2 shows high activity against the studied bacterial strains. This is explained by the formation of hydrogen bonds with the active centers of cellular constituents, disrupting the normal cell structure and causing bacterial death [84]. On the other hand, Wen et al.'s research [83] tested various imidazole derivatives for their antibacterial activities and showed that compounds carrying a smaller alkyl amine motif exhibit moderate antibacterial activities. This is explained by the fact that the lipophilicity and size of the side chain of these derivatives play a crucial role in their antimicrobial activities.

#### 5.1.3 Anticancer activities

Studies on imidazole compounds as anticancer agents have shown that some of these derivatives may exhibit antiproliferative properties and induce apoptosis in cancer cells. However, it is important to note that the anticancer efficacy can depend on various factors, such as the specific chemical structure of the derivative, the specific cell line, and the underlying molecular mechanisms. Table 3 summarizes certain imidazole derivatives along with their anticancer activities.

**Table 3:** Effect of various imidazole derivatives on certain anticancer cell lines.

Compounds	Cell Line	IC_50_ (μM)	Mechanism	Ref.
	Lymphoma Ascites (DLA)	138.50	Not determined	[85]
	Erlich's Ascites Carcinoma (EAC)	31.25	Not determined	
	Lymphoma Ascites (DLA)	102.86	Not determined	[85]
	Erlich's Ascites Carcinoma (EAC)	31.25	Not determined	
	Human melanoma cell lines (A375)	3.5	Anti-proliferative	[86]
	Human melanoma cell lines (M14)	5.6		
	Human melanoma cell lines (RPMI7951)	5.6		
	Human melanoma cell lines (A375)	1.1	Anti-proliferative	[86]
	Human melanoma cell lines (M14)	1.2		
	Human melanoma cell lines (RPMI7951)	3.3		
	Human umbilical vein endothelial cells (HUVECs)	0.4	Selective inhibitor for the HUVECs.	[87]
	Smooth muscle cells (SMCs)	5.5	Activation of p38 signaling pathway.	
	Human myeoloid leukemia cells (HL-60)	0.2	Exhibited excellent inhibitory activity on tumor growth in vivo	[88]
	Human myeoloid leukemia cells (K562)	1		
	Human myeoloid leukemia cells (K562R)	0.9		
	Human prostate carcinoma cells (PC-3)	3.1		
	Human breast carcinoma cells (MCF-7)	10.6		
	Human esophageal carcinoma cells (ECA-109)	3.8		
	Human hepatocarcinoma cells (BEL-7402)	1.2		
	human non-small lung cancer cells (A549)	1.3		
	Human breast cancer cell line (MDA-MB-231)	6.76	Anti-proliferative	[89]
	Human breast cancer cell line (T47D)	9.96		
	Human breast carcinoma cells (MCF-7)	5.71		
	Human lung cancer cell line (A549)	2.29		
	Human colon adenocarcinoma cell line (HT-29)	3.29		
	Human breast cancer cell line (MDA-MB-231)	2.29	Anti-proliferative	[89]
	Human breast cancer cell line (T47D)	5.35		
	Human breast carcinoma cells (MCF-7)	3.46		
	Human lung cancer cell line (A549)	10.6		
	Human colon adenocarcinoma cell line (HT-29)	11.02		

The imidazole derivatives listed in Table 3 exhibit significant anticancer activities against various cancer cell lines. Indeed, imidazole has the potential to overcome the limitations of current clinical drugs such as methotrexate, etoposide, and paclitaxel, which are used as chemotherapeutic agents. Therefore, the imidazole core could be utilized as a chemical structure for new anticancer agents with multiple potential mechanisms of action. In this regard, Sharma et al. [85] studied several imidazole derivatives that showed potent activity with a notable half-maximal inhibitory concentration (IC_50_). The presence of a phenolic group in imidazole compounds significantly affects activity due to their binding capability to cytoplasmic hormone receptors. Furthermore, in the search for more potent and less toxic anticancer agents, several imidazole compounds were synthesized by Xue et al. [88] and tested in vitro. Among them, the compound mentioned in Table 3 exhibited higher activity against breast cancer cells (MCF-7) with an IC_50_ of 10.6 μM compared to doxorubicin. This compound also demonstrated potent anticancer activity, with an IC_50_ of 1.3 μM, against lung cancer cells (A549), and the authors concluded that these derivatives mediate anticancer activity by inducing apoptosis and suppressing cancer cell proliferation. Using in vitro experiments, Kalra et al. [89] investigated twenty-two compounds for their cytotoxicity, and the two compounds mentioned in Table 3 showed cytotoxic activity equal or more potent than docetaxel in a dose-dependent manner.

#### 5.1.4 Advances in preclinical approaches to imidazole-based drug discovery

The imidazole ring represents one of the most important nitrogen heterocycles, widely studied and exploited by the pharmaceutical industry in the search for new drugs. Due to their distinctive structural features and rich electronic environment, drugs containing imidazole rings interact with a variety of therapeutic targets, manifesting a diversity of biological activities. Numerous imidazole-based drugs are commonly used clinically to treat a variety of conditions, presenting significant therapeutic potential. Given their considerable medicinal value, the research and development of drugs containing the imidazole motif remains a dynamic and attractive area of medicinal chemistry. Currently, a substantial number of imidazole-motif compounds are in various stages of clinical trials, all of which have passed extensive preclinical evaluations. Details of parameters such as pharmacokinetics (PK), pharmacodynamics (PD) for each drug class, as well as the IUPAC names of selected drugs, are listed in Table 4.

**Table 4:** Presents the structures, IUPAC names, mode of drug action, study models, Pharmacokinetic (PK) profile, Pharmacodynamic (PD) profile, and the targeted diseases.

Compound structure	IUPAC names	Nature of drug action and target receptor	Study model (s)	Pharmacokinetic (PK) parameters	Targeted Disease (s)	Ref.
	BMS-986260	Potent and selective inhibitor of TGFβR1	Mouse MC38 tumor model	Dose (mg/kg) iv/po = 5/10 Cmax (μM) po = 12.7 T½ (h) iv = 5.7 CL (mL/min/kg) iv = 5.6 Vss (L/kg) iv = 2.4	Clinical candidate as Immuno-oncology agent for the treatment of different types of cancers	[90]
	–	Allosteric inhibitor of RORγt	Acute PD model	CL (mL/min/kg) = 13 T1/2 (h) = 3.7 Vd (L/kg) = 0.6 F % = 35 %	Under clinical investigation for the treatment of autoimmune diseases	[91]
	MRTX1719	Lethal Inhibitor of the PRMT5•MTA Complex	CD-1 mouse model Beagle dog model	Dose (mg/kg) iv/po = 3/30 F % = 80 T½ (h) iv = 1.5 CL (mL/min/kg) iv = 83 Vss (L/kg) iv = 6.3	MTAP deleted Cancers	[92]
	Lanraplenib (GS9876)	Spleen tyrosine kinase Inhibitor	Spontaneous lupus efficacy model	Dose (mg/kg) iv = 1.0 Dose (mg/kg) po = 5.0 CL (L/h/kg) = 1.77 Vss (L/kg) = 2.5 T1/2 (h) = 3.7 F % = 60	Currently under clinical evaluation for the treatment of different autoimmune diseases such as systemic lupus erythematosus (SLE) and Lupus Nephritis (LN)	[93]
	BAY 1895344	Potent, Highly Selective, Orally Available ATR Inhibitor	Rat cancer xenograft model	Dose (mg/kg) = CLbiliary (mL/min/kg) = 1.2 F % = 87 Vss (L/kg) = 1.7 T1/2 (h) = 1.3	Solid tumors and Lymphomas	[94]

**Abbreviations:** CL (Clearance), T_1/2_ (Half Life), PK (Pharmacokinetics), Vss (Steady state volume), F (Bioavailability), TGFβR1 (Transforming growth factor beta receptor 1), RORγt (Retinoic acid-related orphan receptor gamma-t).

Within a broad range of compounds screened through a structure-activity relationship (SAR) study, a robust and selective inhibitor of TGFβR1 named BMS-986260 emerged from a lead compound containing both an imidazole. The primary objective of the mentioned SAR studies was to enhance the efficacy, pharmacokinetic (PK) profile, and solubility profile of the drug candidate, as indicated in Table 4. BMS-986260 proved effective orally in the MC38 mouse tumor model when administered in combination with an anti-programmed cell death protein 1 (anti-PD-1) antibody. However, the drug was found to induce cardiovascular toxicities in preclinical studies due to continuous dosing intervals. To reduce these toxicities, a dose interruption schedule was explored and proved effective. An intermittent dosing schedule every two days for one week in a month provided comparable efficacy. Additionally, BMS-986260 is currently under evaluation as a clinical candidate in immuno-oncology for the treatment of various cancer types [90]. Furthermore, Blomgren et al. [93] revealed GS-9973 (entospletinib) as a selective SYK inhibitor currently undergoing clinical evaluation for hematologic malignancies. GS-9876 (lanraplenib), characterized by human pharmacokinetic properties suitable for once-daily administration and devoid of interactions with proton pump inhibitors (PPIs), is presently undergoing clinical evaluation in multiple autoimmune indications. In 2022, Smith et al. [92] targeted a new synthetic imidazole drug for treating cancers with MTAP gene deletion. Their discovery, the novel candidate drug MRTX1719, acts as a potent and selective binder to the PRMT5•MTA complex, selectively inhibiting PRMT5 activity in cells with MTAP deletion compared to wild-type MTAP cells.

## 6 Conclusion

Tri-substituted and tetra-substituted imidazole constitute an essential heterocyclic system known for their numerous biological activities. On the other hand, they are widely used as a crucial synthesis for the production of biologically active compounds. Various strategies and techniques have been applied to achieve the condensation of these molecules. This review delves into the techniques and development of several synthetic routes for 2,4,5 and 1,2,4,5-imidazole tri or tetra-substituted synthesis, along with various analyses of pharmacological activities (antibacterial, antifungal, anticancer, antimicrobial). In conclusion, it has been observed that tri or tetra-substituted imidazoles demonstrate significant efficiency with a strong potential for new syntheses (by reflux or microwave) and can exhibit various biological activities, necessitating further in-depth research.

## Disclosure statement

A conflict of interest has not been reported, according to the authors.

## Data availability statement

Data used to produce this article are available and listed in the references section.

## CRediT authorship contribution statement

**Abdeljalil Hamdi:** Writing – original draft, Investigation, Formal analysis. **Walid Daoudi:** Writing – review & editing, Validation, Software, Formal analysis. **Mohamed Aaddouz:** Investigation, Formal analysis. **Mohamed Azzouzi:** Investigation, Formal analysis. **Hassan Amhamdi:** Supervision, Resources, Project administration. **Abdellah Elyoussfi:** Validation, Methodology. **Abdelmalik EL. Aatiaoui:** Writing – review & editing, Validation, Supervision, Formal analysis. **Dakeshwar Kumar Verma:** Writing – review & editing, Supervision, Resources. **Mohamed Abboud:** Validation, Supervision, Project administration, Funding acquisition. **M'hamed Ahari:** Validation, Supervision.

## Declaration of competing interest

The authors declare that they have no known competing financial interests or personal relationships that could have appeared to influence the work reported in this paper.

## References

[1] Liu B., Chen H., Cao J., Chen X., Xie J., Shu Y., Yan F., Huang W., Qin T. (2024). Imidazole derivative assisted crystallization for high-efficiency mixed Sn–Pb perovskite solar cells. Adv. Funct. Mater..

[2] Xu W.-B., Li S., Zheng C.-J., Yang Y.-X., Zhang C., Jin C.-H. (2024). Synthesis and evaluation of imidazole derivatives bearing imidazo[2,1-b] [1,3,4]thiadiazole moiety as antibacterial agents. Med. Chem..

[3] Nasrollahzadeh M.S., Eskandarpour V., Maleki M.F., Eisvand F., Mashreghi M., Hadizadeh F., Tayarani-Najaran Z., Ghodsi R. (2024). Design, synthesis and biological evaluation of novel imidazole-based benzamide and hydroxamic acid derivatives as potent histone deacetylase inhibitors and anticancer agents. J. Mol. Struct..

[4] Chaudhury D., Banerjee J., Sharma N., Shrestha N. (2015). Routes of synthesis and biological significances of Imidazole derivatives. World Journal of Pharmaceutical Sciences.

[5] Ahmed R.S., Ali R.A., Ahamed L.S. (2009). Synthesis of new 2, 4, 5-triphenyl imidazole derivatives derived from benzoin and studying their biological activity. Journal of Global Pharma Technology.

[6] Al-Adilee K.J., Jawad S.H., Kyhoiesh H.A.K., Hassan H.M. (2024). Synthesis, characterization, biological applications, and molecular docking studies of some transition metal complexes with azo dye ligand derived from 5-methyl imidazole. J. Mol. Struct..

[7] Al-Adilee K., Kyhoiesh H.A.K. (2017). Preparation and identification of some metal complexes with new heterocyclic azo dye ligand 2-[2−- (1- Hydroxy -4- Chloro phenyl) azo ]- imidazole and their spectral and thermal studies. J. Mol. Struct..

[8] Mahmoud A., Mostafa A., Al-Karmalawy A.A., Zidan A., Abulkhair H.S., Mahmoud S.H., Shehata M., Elhefnawi M.M., Ali M.A. (2021). Telaprevir is a potential drug for repurposing against SARS-CoV-2: computational and in vitro studies. Heliyon.

[9] McCann M., Curran R., Ben-Shoshan M., McKee V., Devereux M., Kavanagh K., Kellett A. (2013). Synthesis, structure and biological activity of silver(I) complexes of substituted imidazoles. Polyhedron.

[10] Eicher T., Hauptmann S., Speicher A. (2013).

[11] Nidhi Rani, Trisubstituted Imidazole Synthesis: A Review - Google Scholar, (n.d.). https://scholar.google.com/scholar?hl=fr&as_sdt=0%2C5&q=Trisubstituted+Imidazole+Synthesis%3A+A+Review&btnG= (accessed February 14, 2021).

[12] Mohammadi Ziarani G., Dashtianeh Z., Shakiba Nahad M., Badiei A. (2015). One-pot synthesis of 1,2,4,5-tetra substituted imidazoles using sulfonic acid functionalized silica (SiO2-Pr-SO3H). Arab. J. Chem..

[13] Chen J., Wang Z., Lu Y., Dalton J.T., Miller D.D., Li W. (2008). Synthesis and antiproliferative activity of imidazole and imidazoline analogs for melanoma. Bioorg. Med. Chem. Lett.

[14] Dominianni S.J., Yen T.T. (2002).

[15] Hung T.-C., Huang C.-C., Meng P.-J., Chuang A., Wu S.-J. (1999). Heavy metals in fish tissues and different species of fish from the southern coast of taiwan. Chem. Ecol..

[16] Soujanya Y., Narahari Sastry G. (2007). Theoretical elucidation of the antioxidant mechanism of 1,3-dihydro-1-methyl-2H-imidazole-2-selenol (MSeI). Tetrahedron Lett..

[17] Jain R., Vangapandu S., Jain M., Kaur N., Singh S., Singh P.P. (2002). Antimalarial activities of ring-substituted bioimidazoles. Bioorg. Med. Chem. Lett.

[18] Niwano Y., Seo A., Kanai K., Hamaguchi H., Uchida K., Yamaguchi H. (1994). Therapeutic efficacy of lanoconazole, a new imidazole antimycotic agent, for experimental cutaneous candidiasis in Guinea pigs. Antimicrob. Agents Chemother..

[19] Il Finar Organic Chemistry - AbeBooks, (n.d.). https://www.abebooks.com/book-search/kw/il-finar-organic-chemistry/(accessed November 25, 2022). (Volume 2).

[20] H. Debus, Ueber die Einwirkung des Ammoniaks auf Glyoxal, Justus Liebigs Ann. Chem. 107 (1858) 199–208. 10.1002/jlac.18581070209..

[21] B. Radziszewski, Ueber die Constitution des Lophins und verwandter Verbindungen, Ber. Dtsch. Chem. Ges. 15 (1882) 1493–1496..

[22] Bourissou D., Guerret O., Gabbaï F.P., Bertrand G. (2000). Stable carbenes. Chem. Rev..

[23] Banothu J., Gali R., Velpula R., Bavantula R. (2017). Brønsted acidic ionic liquid catalyzed an efficient and eco-friendly protocol for the synthesis of 2, 4, 5-trisubstituted-1H-imidazoles under solvent-free conditions. Arab. J. Chem..

[24] Safari J., Khalili S.D., Banitaba S.H. (2010). A novel and an efficient catalyst for one-pot synthesis of 2, 4, 5-trisubstituted imidazoles by using microwave irradiation under solvent-free conditions. J. Chem. Sci..

[25] Abbasov V.M., Marzouk A.A., Mammadov A.M., Kazimova S.Z., Talybov A.H. (2012). Imidazole derivatives, synthesis and biological activity. Processes of Petrochemistry and Oil-Refining.

[26] Free Download Organic Chemistry Vol 2 By I L Finar Third Edition - ChemistryDocs.Com, (n.d.). https://chemistrydocs.com/organic-chemistry-vol-2-by-i-l-finar/(accessed November 26, 2022)..

[27] S.S. Qasim, S.S. Ali, S.K. Ahmed, Research Journal of Pharmaceutical, Biological and Chemical Sciences, (n.d.)..

[28] Nalage S.V., Kalyankar M.B., Patil V.S., Bhosale S.V., Deshmukh S.U., Pawar R.P. (2010). An efficient noncatalytic protocol for the synthesis of trisubstituted imidazole in polyethylene glycol using microwaves. Open Catal. J..

[29] Mala S. (2016). Design, Synthesis, Characterization and Biological Evaluation of Some Novel Anti Tubercular Agents Targeting L, D-Transpeptidase-2.

[30] Sparks R.B., Combs A.P. (2004). Microwave-assisted synthesis of 2,4,5-Triaryl-imidazole; A novel thermally induced N-hydroxyimidazole N−O bond cleavage. Org. Lett..

[31] Steck E.A., Day A.R. (2002).

[32] Sharma V., Khan M.S.Y. (2001). Synthesis of novel tetrahydroimidazole derivatives and studies for their biological properties. Eur. J. Med. Chem..

[33] Heravi M.M., Bakhtiari K., Oskooie H.A., Taheri S. (2007). Synthesis of 2,4,5-triaryl-imidazoles catalyzed by NiCl2·6H2O under heterogeneous system. J. Mol. Catal. Chem..

[34] Sharma S.D., Hazarika P., Konwar D. (2008). An efficient and one-pot synthesis of 2, 4, 5-trisubstituted and 1, 2, 4, 5-tetrasubstituted imidazoles catalyzed by InCl3· 3H2O. Tetrahedron Lett..

[35] Sadeghi B., Mirjalili B.B.F., Hashemi M.M. (2008). BF3·SiO2: an efficient reagent system for the one-pot synthesis of 1,2,4,5-tetrasubstituted imidazoles. Tetrahedron Lett..

[36] Husain A., Drabu S., Kumar N. (2009). Synthesis and biological screening of di-and trisubstituted imidazoles. Acta Pol. Pharm..

[37] Joshi R.S., Mandhane P.G., Shaikh M.U., Kale R.P., Gill C.H. (2010). Potassium dihydrogen phosphate catalyzed one-pot synthesis of 2,4,5-triaryl-1H-imidazoles. Chin. Chem. Lett..

[38] Oliveira E., Baptista R.M.F., Costa S.P.G., Raposo M.M.M., Lodeiro C. (2010). Exploring the emissive properties of new azacrown compounds bearing aryl, furyl, or thienyl moieties: a special case of chelation enhancement of fluorescence upon interaction with Ca2+, Cu2+, or Ni2+. Inorg. Chem..

[39] Shaterian H.R., Ranjbar M. (2011). An environmental friendly approach for the synthesis of highly substituted imidazoles using Brønsted acidic ionic liquid, N-methyl-2-pyrrolidonium hydrogen sulfate, as reusable catalyst. J. Mol. Liq..

[40] Pasha M.A., Nizam A. (2011). p-TSA catalysed efficient synthesis of 1,2,4,5-tetraaryl-imidazoles. J. Saudi Chem. Soc..

[41] Vijesh A.M., Isloor A.M., Telkar S., Peethambar S.K., Rai S., Isloor N. (2011). Synthesis, characterization and antimicrobial studies of some new pyrazole incorporated imidazole derivatives. Eur. J. Med. Chem..

[42] Maleki A., Alirezvani Z., Ghamari N. (2013). The 17th International Electronic Conference on Synthetic Organic Chemistry.

[43] Gharib A., Khorasani BrH., Jahangir M., Roshani M., Bakhtiari L., Mohadeszadeh S. (2014). Synthesis of 2, 4, 5-trisubstituted and 1, 2, 4, 5-tetrasubstituted-1H-imidazole derivatives and or 2, 4, 5-triaryloxazoles using of silica-supported preyssler nanoparticles. Bulg. Chem. Commun..

[44] Sndaroos R., Damavandi S. (2014). Synthesis of 8-aryl-7H-acenaphtho[1,2-d]imidazoles by multicomponent reaction of acenaphthylene-1,2-dione and aromatic aldehydes with ammonium acetate catalyzed by ferric hydrogensulfate. Res. Chem. Intermed..

[45] Subeesh M.S., Shanmugasundaram K., Sunesh C.D., Won Y.S., Choe Y. (2015). Utilization of a phenanthroimidazole based fluorophore in light-emitting electrochemical cells. J. Mater. Chem. C.

[46] Bhat S.U., Naikoo R.A., Tomar R. (2016). One pot synthesis of tetra-substituted imidazole derivatives by condensation reaction using zeolite H-ZSM 22 as a heterogeneous solid acid catalyst. Int. Res. J. Pure Appl. Chem..

[47] Wang J., Xu S., Zhao F., Xia H., Wang Y. (2016). Computational and spectroscopic studies of the imidazole-fused phenanthroline derivatives containing phenyl, naphthyl, and anthryl groups. J. Mol. Struct..

[48] Tavgeniene D., Krucaite G., Baranauskyte U., Wu J.-Z., Su H.-Y., Huang C.-W., Chang C.-H., Grigalevicius S. (2017). Phenanthro[9,10-d]imidazole based new host materials for efficient red phosphorescent OLEDs. Dyes Pigments.

[49] Naureen S., Ijaz F., Munawar M.A., Asif N., Chaudhry F., Ashraf M., Khan M.A., Naureen S., Ijaz F., Munawar M.A., Asif N., Chaudhry F., Ashraf M., Khan M.A. (2017). Synthesis of TETRASUBSTITUTD imidazoles containing indole and their ANTIUREASE and antioxidant activities. J. Chil. Chem. Soc..

[50] Ferreira R.C.M., Costa S.P., Gonçalves H., Belsley M., Raposo M.M.M. (2017). Fluorescent phenanthroimidazoles functionalized with heterocyclic spacers: synthesis, optical chemosensory ability and two-photon absorption (TPA) properties. New J. Chem..

[51] Ravindra M.K., Kumara K., Mahadevan K.M., Naik H.B., Reddy K., Lokanath N.K., Naveen S. (2018). Synthesis, characterization, crystal structure and hirshfeld surface analysis of 4-(1-(4-methoxyphenyl)-4, 5-diphenyl-1H-imidazole-2-yl) phenyl carboxylic acid monohydrate, J. Applicable Chem.

[52] Brahmbhatt H., Molnar M., Pavić V. (2018). Pyrazole nucleus fused tri-substituted imidazole derivatives as antioxidant and antibacterial agents. Karbala International Journal of Modern Science.

[53] Ekbote A., Han S.H., Jadhav T., Mobin S.M., Lee J.Y., Misra R. (2018). Stimuli responsive AIE active positional isomers of phenanthroimidazole as non-doped emitters in OLEDs. J. Mater. Chem. C.

[54] Kula S., Szlapa-Kula A., Kotowicz S., Filapek M., Bujak K., Siwy M., Janeczek H., Maćkowski S., Schab-Balcerzak E. (2018). Phenanthro[9,10-d]imidazole with thiophene rings toward OLEDs application. Dyes Pigments.

[55] Jayabharathi J., Panimozhi S., Thanikachalam V. (2018). Hot exciton transition for organic light-emitting diodes: tailoring excited-state properties and electroluminescence performances of donor–spacer–acceptor molecules. RSC Adv..

[56] Amala S., Rajarajan G., Dhineshkumar E., Doss M.A., Seenivasan M. (2019). Design synthesis and characterization of 2-(4,5-dihydro-2,4,5-triphenylimidazol-1-Yl)-6-methylpyridine:FT-IR, NMR, and computational investigation. AIP Conf. Proc..

[57] Jayabharathi J., Goperundevi G., Thanikachalam V., Panimozhi S. (2019). Regulation of singlet and triplet excitons in a single emission layer: efficient fluorescent/phosphorescent hybrid white organic light-emitting diodes. ACS Omega.

[58] Jayabharathi J., Nethaji P., Thanikachalam V., Ramya R. (2019). Derivatives of cyanonaphthyl-substituted phenanthroimidazole as blue emitters for nondoped organic light-emitting diodes. ACS Omega.

[59] Singh I., Rani R., Luxami V., Paul K. (2019). Synthesis of 5-(4-(1H-phenanthro[9,10-d]imidazole-2-yl)benzylidene)thiazolidine-2,4-dione as promising DNA and serum albumin-binding agents and evaluation of antitumor activity. Eur. J. Med. Chem..

[60] Nipate A.S., Jadhav C.K., Chate A.V., Taur K.S., Gill C.H. (2020). β-Cyclodextrin catalyzed access to fused 1,8-dihydroimidazo[2,3-b]indoles via one-pot multicomponent cascade in aqueous ethanol: supramolecular approach toward sustainability. J. Heterocycl. Chem..

[61] Hasanzadeh F., Behbahani F.K. (2020). Synthesis of 8-aryl-7H-acenaphtho[1,2-d]imidazoles using Fe3O4 NPs@GO@C4H8SO3H as a green and recyclable magnetic nanocatalyst. Russ. J. Org. Chem..

[62] Peng H., Wei Z., Wu L., Li X. (2020). Efficient non-doped blue fluorescent OLEDs based on bipolar phenanthroimidazole-triphenylamine derivatives. Opt. Mater..

[63] Swati D., Burungale (2013). Synthesis of 2, 4, 5- triphenyl imidazole derivatives and biological evaluation for their antibacterial and anti-inflammatory activity. Int. J. Pharmaceut. Sci. Res..

[64] Kula S., Krawczyk P., Filapek M., Maroń A.M. (2021). Influence of N-donor substituents on physicochemical properties of phenanthro[9,10-d]imidazole derivatives. J. Lumin..

[65] Lunt E., Newton C.G., Smith C., Stevens G.P., Stevens M.F., Straw C.G., Walsh R.J., Warren P.J., Fizames C., Lavelle F. (Feb., 1987). J. Med. Chem..

[66] Bhatnagar A., Sharma P.K., Kumar N. (2011). A review on “Imidazoles”: their chemistry and pharmacological potentials. Int J PharmTech Res.

[67] Suzuki F., Kuroda T., Tamura T., Sato S., Ohmori K., Ichikawa S. (2002).

[68] Johnson R.A., Huong S.-M., Huang E.-S. (1999). Inhibitory effect of 4-(4-fluorophenyl)-2-(4-hydroxyphenyl)-5-(4-pyridyl)1H-imidazole on HCMV DNA replication and permissive infection. Antivir. Res..

[69] Brewer M.D., Dorgan R.J.J., Manger B.R., Mamalis P., Webster R.A.B. (2002).

[70] Sumarsiha S., Pratiwia M.D., Ainnia I.N., Sinatriyaa H.R., Soegijantob S., Suciptob T.H., Setyawatia H. (2020). The influence of metal on the performance of 2, 4, 5-triphenylimidazole as an inhibitor of dengue virus replication. Asia Pac. J. Mol. Biol. Biotechnol..

[71] Soni J., Sethiya A., Sahiba N., Agarwal D.K., Agarwal S. (2019). Contemporary progress in the synthetic strategies of imidazole and its biological activities. Curr. Org. Synth..

[72] Puratchikody A., Doble M. (2007). Antinociceptive and antiinflammatory activities and QSAR studies on 2-substituted-4,5-diphenyl-1H-imidazoles. Bioorg. Med. Chem..

[73] Tanigawara Y., Aoyama N., Kita T., Shirakawa K., Komada F., Kasuga M., Okumura K. (1999). CYP2C19 genotype–related efficacy of omeprazole for the treatment of infection caused by Helicobacter pylori. Clinical Pharmacology & Therapeutics.

[74] Nikalje A.P.G., Tiwari S.V., Sarkate A.P., Karnik K.S. (2018). Imidazole-thiazole coupled derivatives as novel lanosterol 14-α demethylase inhibitors: ionic liquid mediated synthesis, biological evaluation and molecular docking study. Med. Chem. Res..

[75] Kidwai M., Mothsra P. (2006). A one-pot synthesis of 1,2,4,5-tetraarylimidazoles using molecular iodine as an efficient catalyst. Tetrahedron Lett..

[76] Ghorbani-Vaghei R., Izadkhah V., Mahmoodi J., Karamian R., Ahmadi Khoei M. (2018). The synthesis of imidazoles and evaluation of their antioxidant and antifungal activities. Monatsh. Chem..

[77] Osmaniye D., Kaya Cavusoglu B., Saglik B.N., Levent S., Acar Cevik U., Atli O., Ozkay Y., Kaplancikli Z.A. (2018). Synthesis and anticandidal activity of new imidazole-chalcones. Molecules.

[78] Husain A., Drabu S., Kumar N., Alam M.M., Bawa S. (2013). Synthesis and biological evaluation of di-and tri-substituted imidazoles as safer anti-inflammatory-antifungal agents. J. Pharm. BioAllied Sci..

[79] Zinad D.S., Mahal A., Shareef O.A. (2020). Antifungal activity and theoretical study of synthesized pyrazole-imidazole hybrids. IOP Conf. Ser. Mater. Sci. Eng..

[80] Abhishek K Jain A.K., Ravichandran V., Sisodiya M., Agrawal R. (2010). Synthesis and antibacterial evaluation of 2–substituted–4,5–diphenyl–N–alkyl imidazole derivatives. Asian Pac. J. Tropical Med..

[81] Valls A., Andreu J.J., Falomir E., Luis S.V., Atrián-Blasco E., Mitchell S.G., Altava B. (2020). Imidazole and imidazolium antibacterial drugs derived from amino acids. Pharmaceuticals.

[82] Atia A.J.K. (2009). Synthesis and antibacterial activities of new metronidazole and imidazole derivatives. Molecules.

[83] Gu W., Qiao C., Wang S.-F., Hao Y., Miao T.-T. (2014). Synthesis and biological evaluation of novel N-substituted 1H-dibenzo[a,c]carbazole derivatives of dehydroabietic acid as potential antimicrobial agents. Bioorg. Med. Chem. Lett.

[84] Japp F.R., Robinson H.H. (1882). Constitution des Lophins und des Amarins. Ber. Dtsch. Chem. Ges..

[85] Sharma G.K., Kumar S., Pathak D. (2010). Synthesis, antibacterial and anticancer activities of some novel imidazoles. Der Pharm. Lett..

[86] Wang Q., Arnst K.E., Wang Y., Kumar G., Ma D., Chen H., Wu Z., Yang J., White S.W., Miller D.D., Li W. (2018). Structural modification of the 3,4,5-trimethoxyphenyl moiety in the tubulin inhibitor VERU-111 leads to improved antiproliferative activities. J. Med. Chem..

[87] Chung K.-H., Hong S.-Y., You H.-J., Park R.-E., Ryu C.-K. (2006). Synthesis and biological evaluation of 5-arylamino-1H-benzo[d]imidazole-4,7-diones as inhibitor of endothelial cell proliferation. Bioorg. Med. Chem..

[88] Xue N., Yang X., Wu R., Chen J., He Q., Yang B., Lu X., Hu Y. (2008). Synthesis and biological evaluation of imidazole-2-one derivatives as potential antitumor agents. Bioorg. Med. Chem..

[89] Kalra S., Joshi G., Kumar M., Arora S., Kaur H., Singh S., Munshi A., Kumar R. (2020). Anticancer potential of some imidazole and fused imidazole derivatives: exploring the mechanism via epidermal growth factor receptor (EGFR) inhibition. RSC Med. Chem..

[90] Velaparthi U., Darne C.P., Warrier J., Liu P., Rahaman H., Augustine-Rauch K., Parrish K., Yang Z., Swanson J., Brown J., Dhar G., Anandam A., Holenarsipur V.K., Palanisamy K., Wautlet B.S., Fereshteh M.P., Lippy J., Tebben A.J., Sheriff S., Ruzanov M., Yan C., Gupta A., Gupta A.K., Vetrichelvan M., Mathur A., Gelman M., Singh R., Kinsella T., Murtaza A., Fargnoli J., Vite G., Borzilleri R.M. (2020). Discovery of BMS-986260, a potent, selective, and orally bioavailable TGFβR1 inhibitor as an immuno-oncology agent. ACS Med. Chem. Lett..

[91] Zhang H., Lapointe B.T., Anthony N., Azevedo R., Cals J., Correll C.C., Daniels M., Deshmukh S., van Eenenaam H., Ferguson H., Hegde L.G., Karstens W.J., Maclean J., Miller J.R., Moy L.Y., Simov V., Nagpal S., Oubrie A., Palte R.L., Parthasarathy G., Sciammetta N., van der Stelt M., Woodhouse J.D., Trotter B.W., Barr K. (2020). Discovery of N-(Indazol-3-yl)piperidine-4-carboxylic acids as RORγt allosteric inhibitors for autoimmune diseases. ACS Med. Chem. Lett..

[92] Smith C.R., Aranda R., Bobinski T.P., Briere D.M., Burns A.C., Christensen J.G., Clarine J., Engstrom L.D., Gunn R.J., Ivetac A., Jean-Baptiste R., Ketcham J.M., Kobayashi M., Kuehler J., Kulyk S., Lawson J.D., Moya K., Olson P., Rahbaek L., Thomas N.C., Wang X., Waters L.M., Marx M.A. (2022). Fragment-based discovery of MRTX1719, a synthetic lethal inhibitor of the PRMT5•MTA complex for the treatment of MTAP-deleted cancers. J. Med. Chem..

[93] Blomgren P., Chandrasekhar J., Di Paolo J.A., Fung W., Geng G., Ip C., Jones R., Kropf J.E., Lansdon E.B., Lee S., Lo J.R., Mitchell S.A., Murray B., Pohlmeyer C., Schmitt A., Suekawa-Pirrone K., Wise S., Xiong J.-M., Xu J., Yu H., Zhao Z., Currie K.S. (2020). Discovery of lanraplenib (GS-9876): a once-daily spleen tyrosine kinase inhibitor for autoimmune diseases. ACS Med. Chem. Lett..

[94] Lücking U., Wortmann L., Wengner A.M., Lefranc J., Lienau P., Briem H., Siemeister G., Bömer U., Denner K., Schäfer M., Koppitz M., Eis K., Bartels F., Bader B., Bone W., Moosmayer D., Holton S.J., Eberspächer U., Grudzinska-Goebel J., Schatz C., Deeg G., Mumberg D., von Nussbaum F. (2020). Damage incorporated: discovery of the potent, highly selective, orally available ATR inhibitor BAY 1895344 with favorable pharmacokinetic properties and promising efficacy in monotherapy and in combination treatments in preclinical tumor models. J. Med. Chem..

